# Immunogenicity and efficacy of a chimpanzee adenovirus-vectored Rift Valley Fever vaccine in mice

**DOI:** 10.1186/1743-422X-10-349

**Published:** 2013-12-05

**Authors:** George M Warimwe, Gema Lorenzo, Elena Lopez-Gil, Arturo Reyes-Sandoval, Matthew G Cottingham, Alexandra J Spencer, Katharine A Collins, Matthew DJ Dicks, Anita Milicic, Amar Lall, Julie Furze, Alison V Turner, Adrian VS Hill, Alejandro Brun, Sarah C Gilbert

**Affiliations:** 1 The Jenner Institute, University of Oxford, Oxford, UK; 2 Centro de Investigación en Sanidad Animal, Instituto Nacional de Investigación Agraria y Alimentaria, Valdeolmos, Madrid, Spain

**Keywords:** Rift Valley Fever, Adenovirus vector, Vaccine

## Abstract

**Background:**

Rift Valley Fever (RVF) is a viral zoonosis that historically affects livestock production and human health in sub-Saharan Africa, though epizootics have also occurred in the Arabian Peninsula. Whilst an effective live-attenuated vaccine is available for livestock, there is currently no licensed human RVF vaccine. Replication-deficient chimpanzee adenovirus (ChAd) vectors are an ideal platform for development of a human RVF vaccine, given the low prevalence of neutralizing antibodies against them in the human population, and their excellent safety and immunogenicity profile in human clinical trials of vaccines against a wide range of pathogens.

**Methods:**

Here, in BALB/c mice, we evaluated the immunogenicity and efficacy of a replication-deficient chimpanzee adenovirus vector, ChAdOx1, encoding the RVF virus envelope glycoproteins, Gn and Gc, which are targets of virus neutralizing antibodies. The ChAdOx1-GnGc vaccine was assessed in comparison to a replication-deficient human adenovirus type 5 vector encoding Gn and Gc (HAdV5-GnGc), a strategy previously shown to confer protective immunity against RVF in mice.

**Results:**

A single immunization with either of the vaccines conferred protection against RVF virus challenge eight weeks post-immunization. Both vaccines elicited RVF virus neutralizing antibody and a robust CD8^+^ T cell response.

**Conclusions:**

Together the results support further development of RVF vaccines based on replication-deficient adenovirus vectors, with ChAdOx1-GnGc being a potential candidate for use in future human clinical trials.

## Background

Rift valley fever (RVF) is one of numerous zoonotic diseases affecting human and livestock health in Africa, and has previously spread to the Arabian Peninsula [1,2]. The disease is caused by a mosquito-borne, negative-stranded RNA virus of the *Bunyaviridae* family, RVF virus, that was first isolated in 1930 from sheep on a Kenyan farm [3]. RVF virus can infect a wide range of domestic and wild animals, but pathology is most severe in sheep where almost 100% mortality and abortion rates occur in newborn lambs and pregnant ewes, respectively [3]. In humans, RVF primarily occurs following close contact with infected animal tissue or body fluids and presents as a mild febrile illness that sometimes progresses to more severe, fatal manifestations such as encephalitis and hemorrhage. Although a highly effective live-attenuated vaccine known as Clone 13 [4] is available for livestock use in RVF-endemic countries, no licensed livestock vaccines are available for use in RVF-free areas such as Europe and there is currently no licensed human RVF vaccine.

Humans and animals recovering from infection with RVF virus develop long-lasting immunity that is attributable to the acquisition of virus-neutralizing antibodies [3,5-8]. These virus-neutralizing antibodies mainly target the Gn and Gc envelope glycoproteins (of which there is only one serotype) encoded in the M segment of the RVF virus genome [9-11]. Subunit vaccines incorporating one or both glycoproteins can induce a virus-neutralizing response that may confer complete protection from experimental RVF viral challenge in rodents and livestock (reviewed in [12]). Thus, development of Gn and Gc-based vaccines utilizing vectors with an established human safety profile could be a promising strategy for a future human RVF vaccine.

Replication-deficient adenovirus vectors have so far been used in human clinical trials of vaccines against *Plasmodium falciparum*[13], human immunodeficiency virus [14], *Mycobacterium tuberculosis*[15], hepatitis C virus [16] and influenza virus [17] in many thousands of adults, children and infants in Europe and Africa, including countries that are prone to frequent RVF epizootics. These studies have highlighted the excellent safety and immunogenicity profile of adenovirus vectors in humans, with similar properties observed in several animal species (including those susceptible to RVF) where adenovirus-vectored vaccines have been tested against multiple diseases [18-27]. Development of an effective adenovirus-vectored RVF vaccine may therefore provide a prophylactic tool that could be used not only in humans, but also in the animal species susceptible to RVF.

To this end, we evaluated the immunogenicity and efficacy of a replication-deficient chimpanzee adenovirus vector, ChAdOx1 [28], encoding the RVF virus glycoproteins Gn and Gc (ChAdOx1-GnGc) in BALB/c mice. The ChAdOx1 vector, unlike the widely tested human adenovirus type 5 (HAdV5) vector, is not affected by significant pre-existing anti-vector immunity that may limit vaccine performance in the human population [28]. Though ChAdOx1 is phylogenetically classified as a *Human adenovirus E*, its serotype is distinct from HAdV5 (a *Human adenovirus C*) [28-30], meaning that anti-vector immunity to HAdV5 cannot dampen the potency of a ChAdOx1-vectored vaccine. Furthermore, ChAdOx1 is currently in clinical development for human influenza and tuberculosis vaccines, making it a good candidate vector for a human RVF vaccine.

We therefore assessed the immunogenicity and efficacy of the ChAdOx1-GnGc vaccine in comparison with a HAdV5 vector encoding Gn and Gc (HAdV5-GnGc), a strategy previously shown to induce protective immunity against RVF in mice [31]. In addition, we examined the effect of two commercially available adjuvants, Matrix-M™ and AddaVax™, on the RVF virus neutralizing antibody response elicited by the ChAdOx1-GnGc and HAdV5-GnGc vaccines. Matrix-M™ is a saponin-based adjuvant developed for human use [32], whereas AddaVax™ is a squalene-based oil-in-water emulsion whose formulation is similar to the MF59® adjuvant licensed for human influenza vaccines [33]. Both these adjuvants were selected for use in this study based on their ability to enhance antibody responses induced by candidate human influenza vaccines [32,34-36].

## Results

### Induction of RVF virus neutralizing antibodies and efficacy against RVF virus challenge

**Figure 1:**
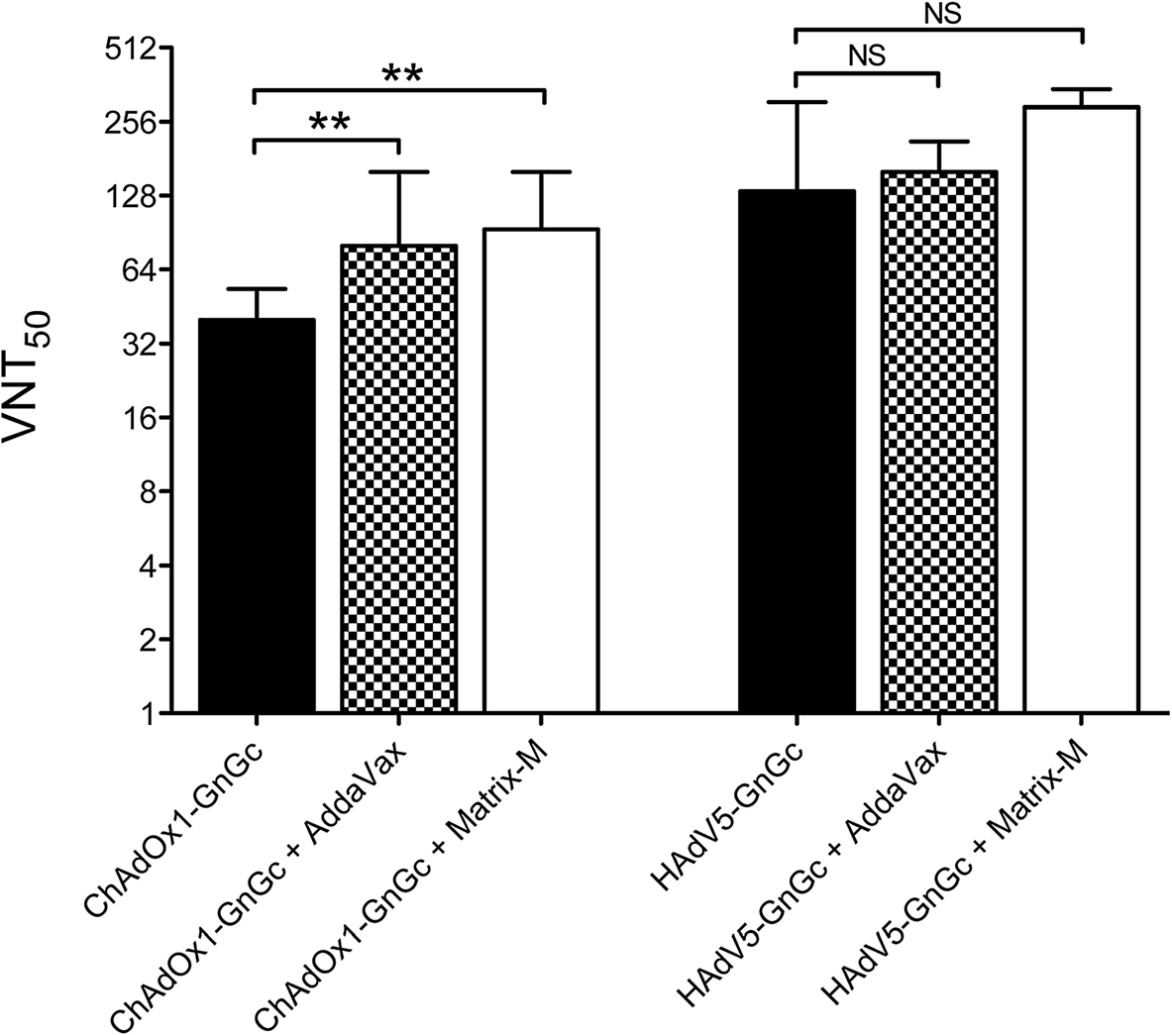
**Induction of virus neutralizing antibodies by ChAdOx1-GnGc and HAdV5-GnGc vaccines.** For each vaccination regimen the virus neutralizing response (VNT_50_), acquired as the antibody titer resulting in 50% reduction of plaque formation relative to cells incubated with RVF virus in the absence of mouse serum, is shown. The data represent medians (bars) and interquartile ranges (error bars) for 14 mice per regimen. The Mann–Whitney U test is used for statistical comparisons between regimens. ** *p* <0.01, NS – not significant.

We first compared RVF virus neutralizing antibody titers between vaccination regimens, measured in sera sampled eight weeks post-vaccination. As shown in Figure 1 both vaccines elicited RVF virus neutralizing antibodies, though, in the absence of adjuvants, mice receiving HAdV5-GnGc mounted higher virus neutralizing titers than those vaccinated with ChAdOx1-GnGc (Mann–Whitney U test *p* = 0.004; Figure 1). However, this difference in immunogenicity was no longer evident when comparisons were made between the HAdV5-GnGc group and the groups receiving ChAdOx1-GnGc in co-administration with either Matrix-M™ or AddaVax™ adjuvant (Kruskal-Wallis test *p* = 0.6).

Both Matrix-M™ and AddaVax™ adjuvant significantly enhanced the RVF virus neutralizing response induced by ChAdOx1-GnGc, but the slight increase in HAdV5-GnGc immunogenicity by either adjuvant did not reach statistical significance (Figure 1). Nevertheless, all ChAdOx1-GnGc and HAdV5-GnGc vaccination regimens conferred protection from clinical disease and mortality following challenge with a lethal dose of the South African 56/74 RVF virus strain (Table 1). In contrast all unvaccinated controls developed clinical illness following RVF viral challenge, with five of six mice succumbing to the infection.

**Table 1:** Efficacy of ChAdOx1-GnGc and HAdV5-GnGc vaccines against RVF virus challenge

**Vaccination regimen**	**Number sick/Total challenged**	**Viraemia post-challenge**
		**(TCID**_**50**_**/ml)**
ChAdOx1-GnGc	0/6	0
ChAdOx1-GnGc + 25μl AddaVax™	0/6	0
ChAdOx1-GnGc + 25μg Matrix-M™	0/6	0
HAdV5-GnGc	0/6	0
HAdV5-GnGc + 25μl AddaVax™	0/6	0
HAdV5-GnGc + 25μg Matrix-M™	0/6	0
Unvaccinated controls	6/6	21380

For each vaccination regimen the number of mice developing clinical signs of RVF following challenge with RVF virus is shown. Viraemia, assessed by RVF virus isolation three days post-challenge, is presented as median TCID50/ml for each regimen.

### Induction of cellular immune responses by ChAdOx1-GnGc and HAdV5-GnGc vaccines

**Figure 2:**
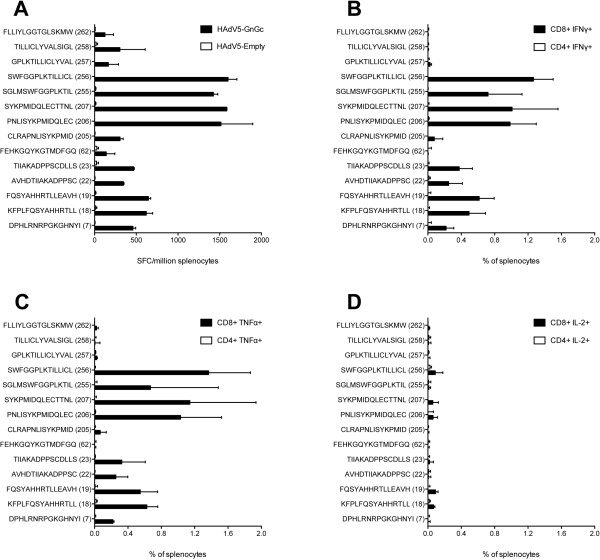
**Cellular responses to overlapping peptides from the Gn and Gc glycoproteins of RVF virus.** Presented is the median (bars) and range (error bars) of number of cells producing IFNγ in an ELISpot assay following restimulation of pooled splenocytes for 18 hours with each of 265 overlapping peptides (each at a final concentration of 5 μg/ml) spanning the entire Gn and Gc glycoprotein from MP-12 RVF virus strain **(A)**. The splenocytes were obtained two weeks post-vaccination with HAdV5-GnGc (N = 5) or HAdV5 lacking a transgenic open reading frame, HAdV5-Empty (N = 5). Only responses exceeding an arbitrary cut-off of >100 SFC/million splenocytes are shown. Responses to all other peptides were below this arbitrary threshold. Numbers in parentheses represent the peptide identification number, with peptides 7, 18, 19, 22, 23 and 62 contained in the Gn glycoprotein and the rest in the Gc glycoprotein. The median frequencies and range of CD8^+^ and CD4^+^ T cells staining positive for IFNγ **(B)**, TNFα **(C)** or IL-2 **(D)** as measured by ICS on splenocytes is also shown.

We next evaluated vaccine-induced T cell responses using overlapping 15mer peptides (N = 265) generated from the Gn and Gc glycoproteins of the MP-12 RVF virus strain. First, the peptides were screened for their ability to re-stimulate interferon gamma (IFNγ) production by splenocytes obtained from mice vaccinated with HAdV5-GnGc as measured by enzyme-linked immunospot assay (ELISpot). In this pilot study, fourteen of the 265 peptides were found to re-stimulate IFNγ ELISpot responses >100 spot forming cells (SFC)/million splenocytes (range, 125 to 1600 SFC/ million splenocytes) (Figure 2A). Responses to all other peptides were below this arbitrary threshold. As shown in Figure 2 several of the fourteen peptides elicited CD8^+^ T cell responses as measured by intracellular cytokine staining assay, but CD4^+^ T cells were barely detectable (Figure 2B-D). Next, a pool of these fourteen peptides, eleven of which encompassed predicted major histocompatibility (MHC) class-I epitopes (Additional file 1: Table S1), was made and used to assess T-cell responses elicited by the ChAdOx1-GnGc and HAdV5-GnGc vaccination regimens.

**Figure 3:**
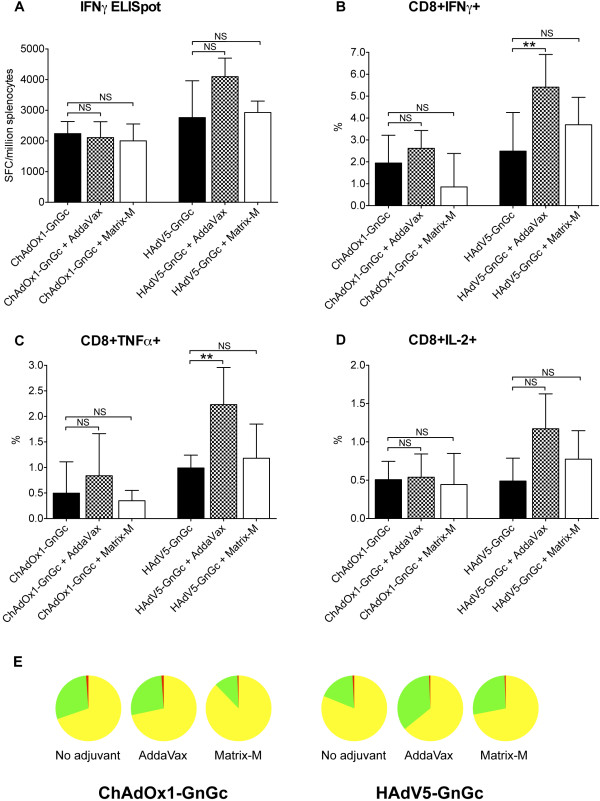
**Induction of T cell responses by ChAdOx1-GnGc and HAdV5-GnGc vaccines.** Presented is a summary of the T cell response elicited by each vaccination regimen as measured by ELISpot assay on splenocytes (**A**; N = 6 mice per regimen) or intracellular cytokine staining of PBMCs (**B** to **E**; N = 14 mice per regimen) two weeks post-vaccination. The bars represent medians and error bars, the interquartile ranges. The Mann–Whitney U test is used for statistical comparisons between regimens. The pie charts in E represent the relative contribution of CD8^+^ T cells staining positive for one cytokine (yellow pie slices), two cytokines (green pie slices) or all three cytokines (red pie slices) to the responses shown in panels **B** to **D**. The raw frequencies of the different CD8^+^ T cell phenotypes are presented in Additional file 1: Figure S2. ** *p* <0.01, NS – not significant.

The frequency of IFNγ spot-forming splenocytes measured by ELISpot was higher among mice receiving HAdV5-GnGc than in those receiving ChAdOx1-GnGc when compared at two weeks (Mann–Whitney U test *p* = 0.03; Figure 3A) or at eight weeks post-vaccination (Mann–Whitney U test *p* = 0.003; Additional file 1: Figure S3). Neither Matrix-M™ nor AddaVax™ significantly enhanced the IFNγ ELISpot responses induced by ChAdOx1-GnGc and HAdV5-GnGc though responses in the HAdV5 plus AddaVax™ regimen were notably higher (Figure 3A and Additional file 1: Figure S3).

Intracellular cytokine staining assay of peripheral blood mononuclear cells (PBMCs) sampled two weeks post-vaccination revealed a robust CD8^+^ T cell response mainly comprising cells staining positive for IFNγ or tumor necrosis factor alpha (TNFα) or both these cytokines and very infrequent interleukin 2 positive (IL-2^+^) cells (Figure 3B-E and Additional file 1: Figure S2). The HAdV5 plus AddaVax™ regimen had the highest frequencies of any of the three CD8^+^ T cell subsets (Figure 3B-D). The predominance of IFNγ and TNFα in the CD8^+^ T cell response was a feature of all vaccination regimens (Figure 3E) and has previously been observed following adenoviral-vectored vaccination of BALB/c mice with recombinant malaria parasite antigens [37].

## Discussion

Adenovirus vectors are ideal for use in vaccines against zoonotic diseases such as RVF, as they have the potential to be deployed in humans and in a wide range of animal species. Replication-deficient chimpanzee adenovirus vectors such as ChAdOx1 are particularly attractive for this purpose given the low prevalence of neutralizing antibodies against them in the human population [28,29,38,39], but they have previously not been utilized for a RVF vaccine.

Here, we have shown that a single immunization with the ChAdOx1-GnGc vaccine confers protection against RVF viral challenge in mice, supporting its further development for use in future human clinical trials and in susceptible animal species. We also confirm previous observations on the induction of protective immunity by HAdV5-vectored RVF viral glycoproteins in mice [31]. Though the high prevalence of neutralizing antibodies against the HAdV5 vector in humans may limit vaccine performance [29,40], the HAdV5-GnGc vaccine could still be used in animals. Indeed, a HAdV5-based vaccine against foot-and-mouth disease has recently been licensed for livestock use in the United States of America [41].

RVF virus neutralizing antibodies have been shown to be sufficient for protection against RVF [12,42] but T cells might also be expected to play a role in immunity through clearance of virus-infected cells. To this end T cell depletion studies have recently found that CD4^+^ T cells, but not CD8^+^ T cells, can influence development of immunity against RVF neuropathology in the C56BL/6 mouse model following infection with an attenuated RVF virus [43]. However, the relative role of different T cell phenotypes in the BALB/c mouse model remains unknown. Here, we find that ChAdOx1-GnGc and HAdV5-GnGc vaccines can readily induce an RVF virus glycoprotein-specific CD8^+^ T cell response in BALB/c mice, as has been observed in a recent study using DNA and modified vaccinia virus Ankara-vectored Gn-Gc vaccines [44]. The robust immunogenicity of both ChAdOx1-GnGc and HAdV5-GnGc for RVF virus neutralizing antibodies and for CD8^+^ T cells presents an opportunity for dissecting the relative contribution of CD8^+^ T cells to protective immunity against RVF in the BALB/c mouse model.

Finally we sought to assess whether two commercially available adjuvants, Matrix-M™ and AddaVax™, could enhance the immunogenicity of ChAdOx1-GnGc and HAdV5-GnGc, with the aim of selecting a vaccine plus adjuvant regimen for evaluation of a vaccine dose-sparing effect in future trials in livestock. Both adjuvants improved the RVF virus neutralizing antibody response induced by ChAdOx1-GnGc, but not HAdV5-GnGc. However, neither significantly enhanced the vaccine-induced T cell response to ChAdOx1-GnGc but co-administration of HAdV5-GnGc with AddaVax™ resulted in an increase in the frequency of CD8^+^ IFNγ^+^ and CD8^+^ TNFα^+^ T cells. These findings may suggest that Matrix-M™ and AddaVax™ could be generally useful for antibody induction by other chimpanzee adenovirus-vectored vaccines, and AddaVax™ for T cell induction by HAdV5-vectored vaccines, but this requires further study. The greatest enhancement in ChAdOx1-GnGc-induced virus neutralizing antibody titers was with Matrix-M™ adjuvant, which is licensed for both human and animal use. Future studies in livestock will be invaluable in determining whether Matrix-M™ adjuvant enhances the magnitude and longevity of the RVF virus neutralizing antibody response induced by ChAdOx1-GnGc, ultimately informing the design of subsequent human clinical trials of ChAdOx1-GnGc.

## Conclusions

In summary our data highlight the potential utility of ChAdOx1 and HAdV5 vectors in vaccines against RVF and provide evidence in support of ChAdOx1-GnGc as a potential candidate for the much-needed human RVF vaccine. Further, both ChAdOx1-GnGc and HAdV5-GnGc hold promise as livestock vaccines and, like most subunit RVF vaccines in development [12], allow differentiation of infected versus vaccinated animals (DIVA) using commercially available DIVA tests that detect immune responses to RVF virus components other than Gn and Gc. Nevertheless, a better idea of the potency of the ChAdOx1-GnGc and HAdV5-GnGc vaccines will only emerge from further studies in target species such as sheep, goats and cattle.

## Methods

### Generation of replication-deficient adenovirus vectored RVF vaccines

The ChAdOx1-GnGc and HAdV5-GnGc genomic clones were prepared by Gateway® recombination (Life Technologies Ltd, UK) between an entry plasmid containing the M segment of the MP-12 RVF virus strain [45], starting from the fourth initiation codon (Genbank accession number DQ380208, bases 411–3614), and an E1- and E3-deleted ChAdOx1 destination plasmid [28] or the E1-and E3-deleted human adenovirus type 5 destination plasmid pAd/PL-DEST™ (Life Technologies Ltd, UK, here termed HAdV5). After viral rescue and propagation in human embryonic kidney 293 cells [46], the resulting replication-deficient ChAdOx1-GnGc and HAdV5-GnGc viruses were purified by CsCl gradient ultracentrifugation.

Both vectors encoded the 1,067 amino acid Gn and Gc RVF viral polyprotein under the control of the human cytomegalovirus major immediate early promoter (Additional file 1: Figure S1). The N terminus of the Gn-Gc polyprotein contained an in-frame fusion of the human tissue plasminogen activator leader sequence, known to enhance transgene expression and immunogenicity [47]. The C terminus of the polyprotein contained a H-2K^d^ restricted CD8^+^ T cell epitope SYIPSAEKI from *Plasmodium berghei* circumsporozoite protein (Pb9) [48] and an anti-V5 monoclonal antibody recognition sequence IPNPLLGLD. Western blotting confirmed expression of the respective antigens with the predicted molecular weight by both vectors (Additional file 1: Figure S1).

### Immunizations and RVF virus challenge

For each vaccination regimen, a total of 20 female BALB/c (H-2^d^) mice (Harlan, UK) aged 6–8 weeks old were immunized with 1 × 10^8^ infectious units of the respective vector in phosphate-buffered saline (PBS). These were either administered alone or co-administered with one of two adjuvants, AddaVax™ (InvivoGen, USA, used at 25 μl per mouse) and Matrix-M™ (Isconova, Sweden, used at 25 μg per mouse). All immunizations were intramuscular and were performed under isofluorane anesthesia in a total volume of 50 μl administered to the right posterior tibialis muscle.

Six vaccination regimens were evaluated in this study: i) ChAdOx1-GnGc without adjuvant, ii) ChAdOx1-GnGc plus Matrix-M™ adjuvant, iii) ChAdOx1-GnGc plus AddaVax™ adjuvant, iv) HAdV5-GnGc without adjuvant, v) HAdV5-GnGc plus Matrix-M™ adjuvant and vi) HAdV5-GnGc plus AddaVax™ adjuvant.

Sampling for immunological assays was done as follows. Two weeks post-vaccination, blood samples were taken from eight mice per regimen for intracellular cytokine staining (ICS) assays on peripheral blood mononuclear cells (PBMCs) as described [49]. A further six mice were culled at this time point and spleens and blood were harvested for interferon gamma (IFNγ) enzyme-linked immunospot assay (ELISpot) on splenocytes [50] and ICS assays on PBMCs, respectively. Eight weeks post-vaccination, blood samples were taken from all remaining mice (14 per regimen) and eight mice culled per regimen for IFNγ ELISpot on splenocytes. After another 48 hours the remaining mice (6 mice per regimen), together with an additional group of six unvaccinated BALB/c mice, were all challenged with 1 × 10^3^ plaque-forming units (pfu) of the South African RVF virus strain 56/74 via the intraperitoneal route as described [44].

The primary endpoint for efficacy estimation was the development of clinical signs of illness including ruffled fur, hunched posture and reduced activity, as described [51], and this was monitored daily over 21 days. We also assessed viraemia and mortality post-challenge as secondary endpoints. For assessment of peak viraemia ~50 μl of blood was sampled at days 3 and 6 post-challenge and used for virus isolation. All surviving mice were culled after 21 days of follow-up.

All animal procedures were conducted in accordance with the United Kingdom Animals (Scientific Procedures) Act Project Licence (PPL30/2889) and were approved by the University of Oxford Animal Care and Ethical Review Committee and the Centro de Investigación en Sanidad Animal, Instituto Nacional de Investigación Agraria y Alimentaria (CISA-INIA) Committees on Biosafety and Ethics of Animal Experimentation (permits CBS2012/017 and CEEA2012/14) in accordance with regulatory guidelines from the European Community Council Directive 86/609/EEC.

### Assessment of RVF virus neutralizing antibodies

The titer of vaccine-induced RVF virus-neutralizing antibodies was measured as previously described [44]. Briefly, sera were first heat-inactivated at 56°C for 30 minutes and two-fold serial dilutions of each made in Dulbecco’s Modified Eagle Medium (DMEM) containing 2% fetal bovine serum. Fifty microliters of each diluted serum was mixed with an equal volume of medium containing 1200 pfu of the MP-12 RVF virus strain and incubated for 1 hour at 37°C. This serum-virus mixture was then transferred onto 96-well plates containing Vero cell monolayers and incubated at 37°C in the presence of 5% CO_2_. After 72 hours the cells were fixed and stained in a solution containing 10% formaldehyde and 2% crystal violet in PBS. Plaque formation was then scored and neutralization titer defined as the highest serum dilution at which plaque formation was reduced by 50% relative to that in cells incubated with MP-12 RVF virus only. The assays were performed in triplicate and scored by an operator blinded to vaccination regimen.

### Assessment of T cell responses to RVF viral glycoproteins

RVF viral glycoprotein-specific T cells were measured by IFNγ ELISpot assay on splenocytes [50] and ICS on PBMCs [49]. Our strategy involved: 1) pre-screening of 265 overlapping 15mer peptides (overlapping by 11 amino acids) spanning the Gn and Gc glycoproteins from the MP-12 RVF virus by IFNγ ELISpot on splenocytes from mice vaccinated with HAdV5-GnGc, 2) preparation of a peptide pool composed of peptides found to re-stimulate >100 spot-forming cells (SFC)/million splenocytes, and 3) use of the peptide pool for IFNγ ELISpot and ICS assays on splenocytes and PBMCs from mice vaccinated with the ChAdOx1-GnGc and HAdV5-GnGc regimens considered here. The peptides were synthesized in a PepSet™ format (Mimotopes, UK) to ~70% purity using the Gn and Gc sequence from the MP-12 RVF virus strain (Genbank accession number DQ380208, bases 411–3614). The stock peptides were reconstituted in 100% dimethyl sulfoxide and cell culture media (DMEM containing 10% fetal calf serum) used to further dilute each peptide for use at a final concentration of 5 μg/ml in all assays. For IFNγ ELISpot assays peptide re-stimulation of splenocytes was done for 18 hours [50]. For ICS assays, peptide re-stimulation of PBMCs was done for 5 hours and the frequency of cells staining positive for IFNγ, tumor necrosis factor alpha (TNFα) or interleukin 2 (IL-2) measured by flow cytometry as described [49].

### Statistical analysis

All analyses were performed using GraphPad PRISM® version 5.0 with alpha = 0.05. For each vaccine, the Mann–Whitney U test was used to assess the effect of co-administration with either Matrix-M™ or AddaVax™ adjuvant on the induced antibody and T cell response. The Kruskal-Wallis test was used for univariate comparisons involving more than two regimens.

## Abbreviations

RVF: Rift valley fever; IFNγ: Interferon gamma; TNFα: Tumor necrosis factor alpha; IL-2: Interleukin 2; PBMC: Peripheral blood mononuclear cells; ICS: Intracellular cytokine staining; ELISpot: Enzyme-linked immunospot; DMEM: Dulbecco’s modified eagle medium.

## Competing interests

MDJD, MGC, SCG and AVSH are named inventors on a patent application describing the ChAdOx1 vector (GB Patent application number 1108879.6). All other authors declare that they have no competing interests.

## Authors’ contributions

GMW, MGC, MDJD and AT designed and constructed the vaccines. GMW, AR, AM, AL, JF, EL, GL, AJS, KC and AB performed animal studies, western blots, immunoassays and epitope prediction. SGC and AVSH provided overall guidance in project design and experiments. GMW wrote the manuscript with critical input from all authors. All authors read and approved the final manuscript.

## Supplementary Material

Additional file 1: Figure S1 Vaccine design and confirmation of transgene expression. **Figure S2.** Cytokine profiling of vaccine-induced CD8+ T cells. **Figure S3.** IFNγ ELISpot responses at eight weeks post-vaccination. **Table S1.** Software prediction of epitopes within immunodominant peptides. Click here for file
