# Supplementary material for: Immunogenicity and efficacy of a chimpanzee adenovirus-vectored Rift Valley Fever vaccine in mice
Source: Virol J. 2013 Dec 5;10:349. doi: 10.1186/1743-422X-10-349 (PMC4235025; doi:10.1186/1743-422X-10-349)
Supplement: Additional file 1: Figure S1 — Vaccine design and confirmation of transgene expression. Figure S2. Cytokine profiling of vaccine-induced CD8+ T cells. Figure S3. IFNγ ELISpot responses at eight weeks post-vaccination. Table S1. Software prediction of epitopes within immunodominant peptides. [file 1743-422X-10-349-S1.pdf]

Figure S1: Vaccine design and confirmation of transgene expression

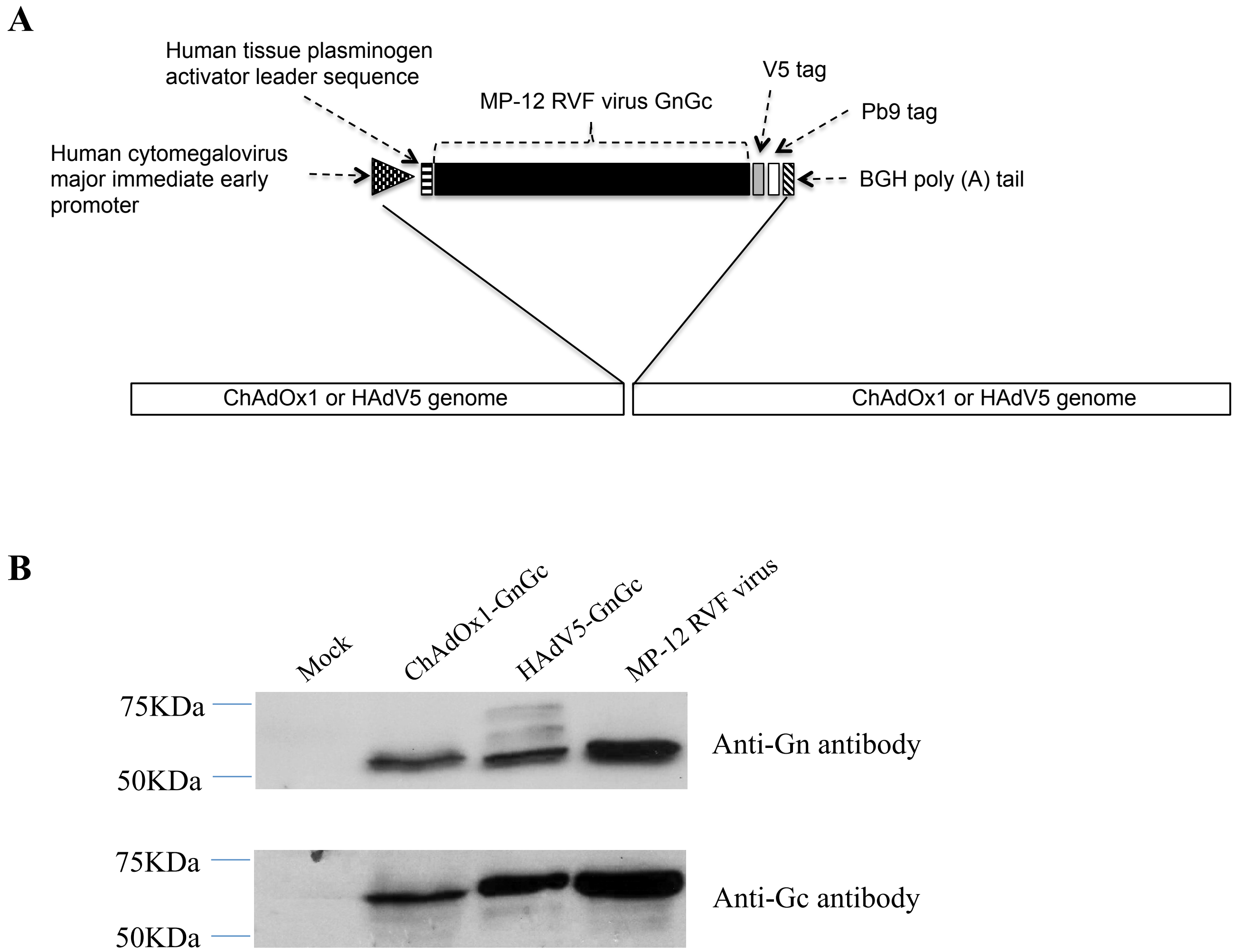

Presented in (A) is a schematic of the vaccine design strategy used in this study. The E1-and E3-deleted ChAdOx1 and HAdV5 vectors each encoded the MP-12 RVF virus M segment (starting from the fourth initiation codon; see Methods) under the control of the human cytomegalovirus major immediate early promoter. The human tissue plasminogen activator leader sequence, V5 and Pb9 tags are as described in the methods. The BGH (bovine growth hormone) polyadenylation signal for termination of protein expression is also shown. In (B) HEK293A cells were infected with ChAdOx1-GnGc, HAdV5-GnGc or MP-12 RVF virus and expression of Gn and Gc glycoproteins assessed by western blot of cell extracts harvested after 48 hours. Cells incubated with culture media alone (“Mock”) were

used as a negative control. A mouse anti-Gn monoclonal antibody and rabbit anti-Gc polyclonal serum were used for the detection of Gn and Gc as described [1].

Figure S2: Cytokine profiling of vaccine-induced CD8+ T cells

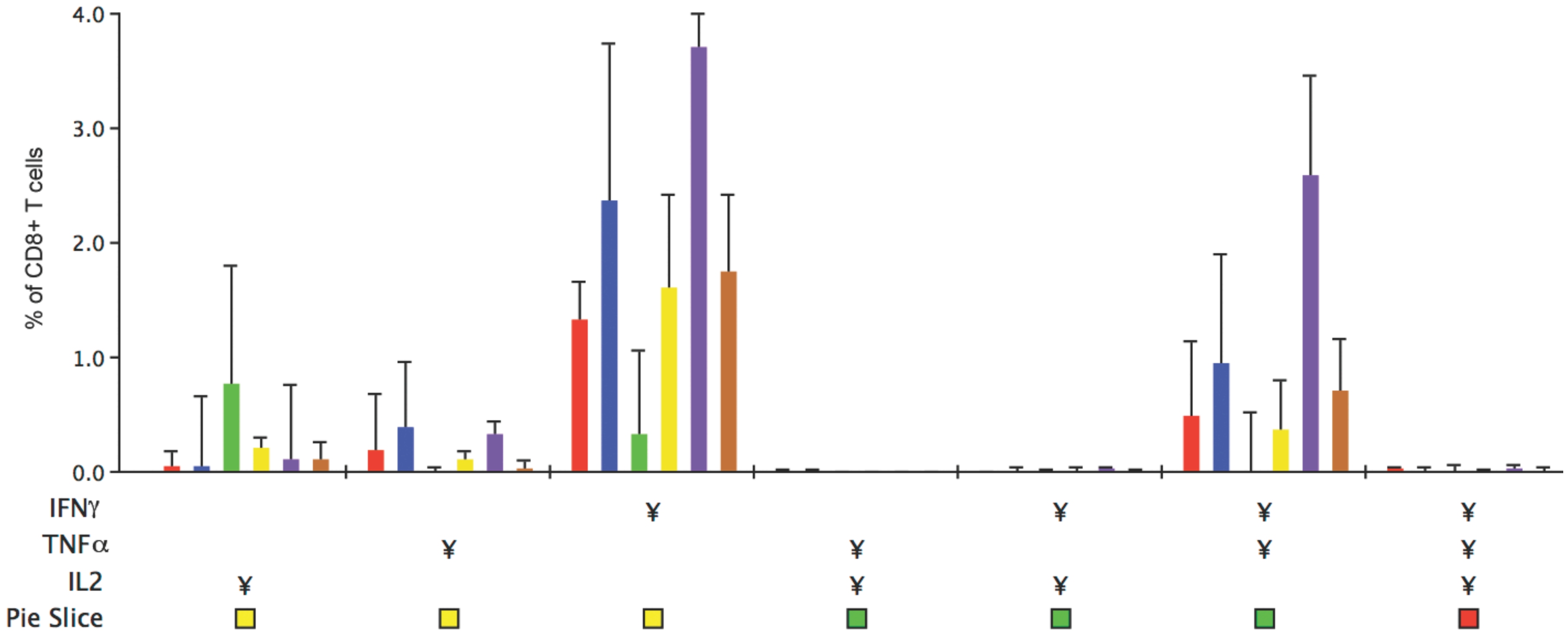

Presented are the median frequencies and interquartile ranges of CD8+ T cell phenotypes detected by intracellular cytokine staining and flow cytometry of peptide-stimulated PBMCs from mice in each vaccination regimen (N = 6 per regimen). For each of the seven possible patterns of cytokine production (indicated by “Pie slice” and “¥” on the legend) the data are ordered by vaccination regimen from left to right as follows: ChAdOx1-GnGc without adjuvant, ChAdOx1-GnGc plus AddaVax™, ChAdOx1-GnGc plus Matrix-M™, HAdV5-GnGc without adjuvant, HAdV5-GnGc plus AddaVax™ and HAdV5-GnGc plus Matrix-M™, respectively. The pie slice colors correspond to those in Figure 3E.

Figure S3: IFNγ ELISpot responses at eight weeks post-vaccination

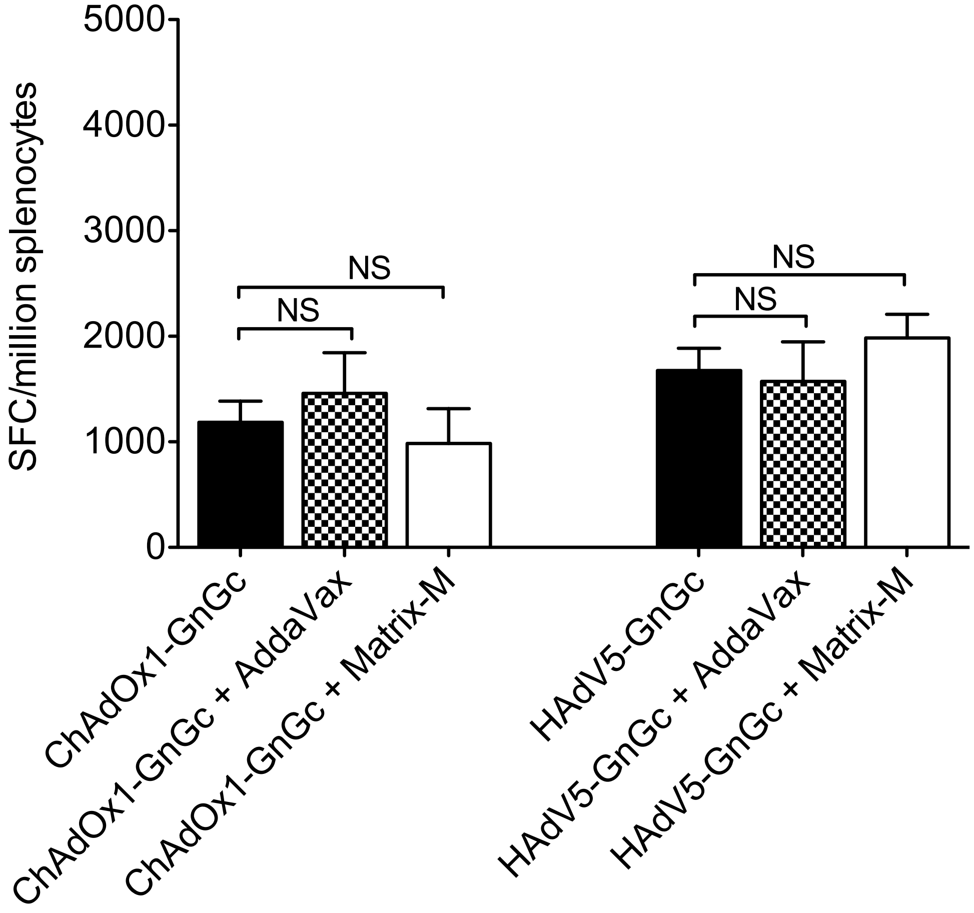

Presented are the median IFNγ ELISpot responses among mice in each of the six vaccination regimens (N=8 mice per regimen). The error bars represent interquartile ranges. The Mann-Whitney U test is used for statistical comparisons between regimens. ** p <0.01, NS – not significant.

Table S1: Software prediction of epitopes within immunodominant peptides

Source peptide	Predicted epitope	Predicted IC50 (nM)	Allele
7	RPGKGHNY	138.35	H-2L^d
18, 19	SYAHHRTLL[1]	7.90	H-2K^d
22, 23, 62	None identified	-	-
205	APNLISYKPM	54.39	H-2L^d
206, 207	SYKPMIDQL[2]	4.71	H-2K^d
255	GGPLKTIL	331.05	H-2D^d
255, 256	SWFGGPLKTI	76.96	H-2K^d
256	GGPLKTILL[3]	241.84	H-2D^d
256, 257	GPLKTILL	126.24	H-2L^d
258	LYVALSIGL	304.91	H-2K^d
262	IYLGGTGL	38.49	H-2K^d

Presented are predicted epitopes and affinities (shown as IC50 in nM) within each of the fourteen peptides presented in Figure S1. For each mouse haplotype and source peptide the predicted epitope with the highest affinity (epitope with the lowest predicted IC50) is shown. The predictions were done using NetMHCpan 2.4 Server (http://www.cbs.dtu.dk/services/NetMHCpan/) and only epitopes with a predicted IC50 <500nM were considered. None of the predicted epitopes within peptides 22, 23 and 62 met this criterion. Previously published epitopes are indicated: [1](2) and [2,3] (1)

REFERENCES

1. **Lopez-Gil E, Lorenzo G, Hevia E, Borrego B, Eiden M, Groschup M, Gilbert SC, Brun A.** 2013. A Single Immunization with MVA Expressing GnGc Glycoproteins Promotes Epitope-specific CD8+-T Cell Activation and Protects Immune-competent Mice against a Lethal RVFV Infection. PLoS neglected tropical diseases **7:**e2309.
2. **Bhardwaj N, Heise MT, Ross TM.** 2010. Vaccination with DNA plasmids expressing Gn coupled to C3d or alphavirus replicons expressing gn protects mice against Rift Valley fever virus. PLoS neglected tropical diseases **4:**e725.
